# Workings of the human spirit in palliative care situations: a consensus model from the Chaplaincy Research Consortium

**DOI:** 10.1186/s12904-015-0005-3

**Published:** 2015-06-02

**Authors:** Linda Emanuel, George Handzo, George Grant, Kevin Massey, Angelika Zollfrank, Diana Wilke, Richard Powell, Walter Smith, Kenneth Pargament

**Affiliations:** Research and Education, HealthCare Chaplaincy Network, 65 Broadway, 12th Floor, 10006-2503 New York, NY USA; Spiritual Health, Emory Healthcare, Emory University, Atlanta, Georgia USA; Mission and Spiritual Care, Advocate Lutheran General Hospital, Chicago, Illinois USA; Clinical Pastoral Education, Yale-New Haven Hospital, New Haven, CT USA; Geriatric Medicine and Buehler Center on Aging, Health & Society, Feinberg School of Medicine, Northwestern University, Chicago, USA; Center of Excellence for End-of-Life Transition Research, Department of Biobehavioral Health Science, College of Nursing, University of Illinois at Chicago, Illinois, USA; Global Health Researcher, Nairobi, Kenya; Psychology, Bowling Green State University, Bowling Green, 43403 Ohio, USA

**Keywords:** Human spirituality, Model, Palliative care, Research, Chaplaincy

## Abstract

**Background:**

Chaplaincy is a relatively new discipline in medicine that provides for care of the human spirit in healthcare contexts for people of all worldviews. Studies indicate wide appreciation for its importance, yet empirical research is limited. Our purpose is to create a model of human spiritual processes and needs in palliative care situations so that researchers can locate their hypotheses in a common model which will evolve with relevant findings.

**Methods:**

The Model Building Subgroup worked with the Chaplaincy Research Consortium as part of a larger Templeton Foundation funded project to enhance research in the area. It met with members for an hour on three successive occasions over three years and exchanged drafts for open comment between meetings. All members of the Subgroup agreed on the final draft.

**Results:**

The model uses modestly adapted existing definitions and models. It describes the human experience of spirituality during serious illness in three renditions: visual, mathematical, and verbal so that researchers can use whichever is applicable. The visual rendition has four domains: spiritual, psychological, physical and social with process arrows and permeable boundaries between all areas. The mathematical rendition has the same four factors and is rendered as an integral equation, corresponding to an integrative function postulated for the human spirit. In both renditions, the model is notable in its allowance for direct spiritual experience and a domain or factor in its own right, not only experience that is created through the others. The model does not describe anything beyond the human experience. The verbal rendition builds on existing work to describe the processes of the human spirit, relating it to the four domains or factors.

**Conclusions:**

A consensus model of the human spirit to generate hypotheses and evolve based on data has been delineated. Implications of the model for how the human spirit functions and how the chaplain can care for the patient or family caregiver’s spiritual coping and well-being are discussed. The next step is to generate researchable hypotheses, results of research from which will give insight into the human spirit and guidance to chaplains caring for it.

## Introduction

Our purpose is to create a model of human spiritual processes and needs in palliative care situations so that researchers in spiritual aspects of palliative care can locate their hypotheses in a common model. This model is intended as one that will evolve with each relevant research finding.

Our approach is consensus based; that is, all members felt they could live with the content although not all would have stated everything exactly as it is in their own words. The Chaplaincy Research Consortium is convened by the HealthCare Chaplaincy and is a community of researchers and scholars. The group’s members were all involved in activities of a three year John Templeton Foundation grant to foster palliative care chaplaincy research.

We use established definitions, established fields, and published works as much as possible and aim to be consistent with understandings of a full range of spiritual and philosophical traditions. We aspire to describing a model within which everyone can identify their experience and tradition in. Our group is diverse but not fully representative of the extent of human perspectives. We represent chaplaincy from a range of faith traditions and none, and in terms of disciplines we have backgrounds in medicine, nursing, psychology, social sciences, and both quantitative and qualitative research. But our group and our work are both dynamic entities and we welcome contributions from those with a better understanding of approaches we do not represent.

Recognizing that models can be built from empirical [1] or conceptual foundations, we wish to be clear that ours is conceptual. While it is built on the insights of experienced professionals, it is not data-driven. Building on existing works that include the spiritual dimension of human experience to create what has been called the biospsychosocialspiritual model, [2] its purpose is to facilitate the generation of testable hypotheses which will, in turn, create data where none currently exists. A future paper will set out some of these hypotheses.

Our focus is the human spirit rather than any specific philosophical or religious framework about the divine. We do not seek to describe the sacred, the divine, or forces of a transcendent or cosmic nature, but rather the human relationship to such ultimate reality. Perhaps analogously to small particle physicists and astronomers, we are interested in the impact on what is knowable since we cannot describe the unknowable. In this case what is knowable is the reported human spiritual experience; we seek to describe a model of its workings. We seek to model, so we can eventually measure, what people experience spiritually.

Our assumption is that the human spirit has essential commonalities across social and religious groups and across human attributes, developmental stages, and types of disability, and we are eager to test the model when applied for any human being in whatever state and whatever philosophical or religious worldview he or she operates in. Recognizing the variety of spiritual experiences, the model of human spiritual workings that we seek to create is one of commonality rather than uniformity, inclusion rather than exclusion, and tolerance rather than skepticism.

Our primary goal is not to resolve ultimate or controversial questions. Instead, it is to enable the asking of empirically answerable questions. We propose a model that generates researchable hypotheses and that evolves according to the findings. It is ‘a place to start’. It is a simple model. It has three main renditions: an overarching concept that is described as a visual diagram of components; a mathematical correlate of that which may help to generate experimental designs; and a narrative description of process model which identifies types of interaction between components.

Our expectation is that this model will primarily help chaplaincy research, but we understand that care of the human spirit is provided by many categories of people [3].

## Definitions

To help clarify what our model describes, we have adopted definitions for some key words.

### Spirituality

Starting with the consensus definition of spirituality created by the Archstone Foundation funded group in 2009, [4] we found it helpful to simplify it and to adjust it by describing the connectedness that we assume the human spirit has to the sacred [5]. We agree that the diverse types of connectedness offered by the original definition – to the moment, to self, to others, to nature, and the significant – are important and can be spiritual, but prefer to define sacred in a way that encompasses these possibilities for spiritual connectedness. With this definition, we hope to distinguish the spiritual from the psychological and social aspects of human experience. We recognize the often intimate overlap between psychological and spiritual and between social and spiritual experience but chose to make the word ‘sacred’ central in order to help identify which components of experiences and needs are spiritual.

We define spirituality as: ‘the aspect of individuals that seeks and perceives significance and experiences connectedness to the sacred’.

### Experiences of sacredness

People from all religions and those who eschew religion and spirituality all describe a sense of awe, greatness and significance (or smallness and insignificance by contrast), preciousness, presence, being in a special place, being transported, time standing still, beauty, aliveness, and love. People describe this experience similarly even though the context and its specifics are different. Perhaps it occurred when holding a new born child, engaging in or watching a dance, engaging in intimate relationship, laughing, making eye contact, appreciating the natural world, riding a motorcycle, playing or hearing or composing music, going deep in prayer or meditation, cooking, running, flying a plane, or making or appreciating art. The list is endless. Perhaps a sense of the sacred occurs often, perhaps rarely; but it seems to occur for everyone. The occurrences are invested with a sense of ultimate importance and profound value. Sometimes, the importance and value of an experience of sacredness can leave the person feeling transformed and great struggles may be dissolved or puzzles answered. Even those who identify themselves as not spiritual have some of these experiences [6,7].

We define experience of the sacred as feeling connected to or aware of the unknowable, the infinite, immanent or transcendent in a way that creates awe, and seems to be precious, and connected to that which enlivens.

### Existential

The word existential has acquired different connotations depending on the discipline or other social context in which it is used. For chaplains, existential tends to connote something akin to the way it was used by Tillich, Heidegger, Hegel, Buber, Frankel and others. In this extensive, scholarly dialogue, it was asserted that understanding human existence requires more than the prior logical systems or dimensions of understanding. For palliative care physicians, nurses, and non-chaplain medical researchers, existential tends to connote something to do with facing mortality, which is often referred to in the context of describing a patient’s ‘existential crisis’ or conversely his or her ‘existential equanimity’ Or ‘existential maturity [8]’.

Whilst accepting the importance of the way the term has been used by theologians, nonetheless in the medical culture, it is often used in a practical way to denote something to do with needs that a person has when confronting the limits of his or her own existence. The view in the medical culture is something like this: since everyone exists and has to deal with mortality, everyone engages existential questions; so spiritual care is needed and has to work for everyone. This is considered to be true for people whether they identify with being religious, or spiritual but not religious, or understand themselves as not at all religious or spiritual. This is important since the spectrum of human experience is broad. The group that is gathering larger numbers in the US according to recent survey is that in which people identify themselves as not religiously affiliated; in 2012 this group included about 20% of the population in the US. However, they appear to be spiritual if not religious; one third describe themselves as spiritual but not religious, over a half feel a deep connection with nature, and over two thirds say they believe in God [9].

The Model Building Subgroup of the Chaplaincy Research Consortium defines spirituality and sacredness with the intent to include all people. So to be consistent, we use the term existential to refer to the human experience of existence in its most ultimate sense.

## Precepts

We base our model on three fundamental precepts.

### The greater existence

First, this model assumes a greater existence than that of humans that we interact with. However, it does not adopt one understanding or another of what the greater world of existence is. We use many words in an effort to indicate that different traditions and approaches can find or place their own name, including by using a name that observes the inability to name, to indicate a connectedness, the cosmos, or a higher power. Some will comfortably use the word God while others will use the word Nirvana, Universe, Love, Nature, Humanity, and so on. We hope that by using many words we can indicate the attributes that traditions have sufficiently in common that we know we are talking about the same thing that the human spirit experiences or relates to or is part of, but we deliberately avoid any definitions for this.

### Connectedness

A second precept is inherent in our definition of spirituality. That is, that humans have a fundamental life connection between what’s ‘in there’ with what’s ‘out there’ that makes a difference to his or her spiritual well being. Our model does not specify what it is that connects us all or what the greater existence is, but the model depicts it as an ultimate phenomenon or reality that is a determinant of humans’ spiritual experience. For some, that connectedness occurs best with solitude, for others with intimate bonds with another, yet others with institutions and rituals, and for most it is sometimes one, sometimes another, and often a blend.

We accept that the nature of that connection for an individual is analogous to the state of being in other spheres of life that may be the necessary features of physical existence (e.g. water), or of psychological well being (e.g. a loving parent), or of social existence (e.g. relationships). While variations in need appear to exist among people, when that aspect of life is awry it causes suffering; a uniquely spiritual suffering.

Evidence for the precept is found in many places. It is explicit in all sacred texts, and in the deep folk and literary narratives and the works of great philosophers from every civilization. Its experience is evidenced in surveys and focus groups. It is prominent in the foundational definition in palliative care of ‘total pain’ by Cecily Saunders which included suffering in all four domains of human experience, namely physical, social, psychological, and spiritual. It is described by clinicians as a distinct type of need for sacred connectedness [10].

Although our model makes this a precept, we also note that in doing so, we open it to empirical research. A well designed and conducted study of the human spiritual experience that found evidence for spiritual experience without such a fundamental need would result in a modification or rejection of our model.

### Human mediators of spiritual experience

The third precept is about humans. In noting that our model does not attempt to describe what is ‘out there’ but only the human dimension, we assume that there is a human substrate which mediates spiritual experiences.

Our model does not specify location. Based on some evidence and experience, it does note a closer connection between psychological and spiritual than between either physical or social and spiritual domains, whilst noting that all spheres interact [11,12]. It does allow for measuring some impacts of spiritual experience using biophysical measures. Importantly, it seeks to allow for hypotheses and future elucidation of the substrate and its workings.

A primary purpose of chaplaincy work is indicated by this fundamental need to connect with what’s ‘out there’ or ‘in there’. The chaplain or other spiritual care provider fosters the person’s ability to connect in a way that brings the person peace or spiritual wellness; a sense of being that allows a person to say, in a highly personal yet expansive way: ‘it is well with the universe’.

The analogue to the primary purposes of other health professionals is straight forward. In the same way that the primary purpose of the Western physician is to engineer the outcome for a patient that allows him or her to say ‘I feel well in my body’, and the primary purpose of mental health professional is to help a person reach the state when she or he feels happy, well, and balanced in the personal sphere, and the eventual purpose of a social worker is to help usher in a sense of wellbeing in society, so too is it the purpose of the chaplain to achieve this state in the spiritual domain.

## Why we care about the nature of the workings of the human spirit

The model is not about care of the spirit *per se*. But it is for the purpose of research about the ways of the human spirit, and the purpose of having a model is to lead to understanding how chaplains and others can best care for the human spirit. In order for chaplains to help a person reach a state of spiritual wellbeing, especially when that person is facing the ultimate challenge to his or her existence due to illness, it is necessary to understand the workings of the human spirit. For instance, in conversation about what chaplains do that works for people, chaplains in our group noted that their patients say, about colleagues who have visited before them, things like: ‘I don’t know who it was or what s/he did, but I felt better’. We want to know what can be learned about what ‘felt better’ entailed or about the kind of listening or presence or ritual or other way of being or doing that created the experience of feeling better. Our hope is that a model will help refine the questions so that well designed research can answer them.

Resistance to considering the workings of the human spirit in analogous ways to anatomy and physiology, psychodynamics, and social theory is understandable. There is a sense that the spirit is mysterious and should not be ‘unpacked’ or defined in a reductionist manner.

However, we note that it is what is ‘out there’ that we cannot name or define that is mysterious and beyond our ability to know. It is not human beings and our workings that are beyond our ability to know. If we feel it, we can measure it. Understanding our human workings is the basis of helpful action. We also note that people all seem to have their own ways of understanding these workings; these understandings are, in essence, a personal model. So, it seems that it is impossible or at least very difficult to operate without some kind of internal model with which to understand what happens. This group takes the view that since the models in our minds define our thinking, including for chaplains, it would be preferable to develop a model that is open and available, well examined and evolvable. In this way, the community of researchers can evolve related understandings that practitioners can use.

A model of the workings of the human spirit should not aspire to be the final word on what the human spirit is. A model can never be what it describes, and descriptions must be updated or adjusted based on gathering evidence. The outcome we seek for the HealthCare Chaplaincy Model of human spirituality is that it grounds and generates hypotheses for researchers whose findings then alter the model accordingly.

The model should allow for particulars but not be particular. That is, if various groups, religiously or otherwise defined, adapt the model for their more specific understandings, we would regard that as a successful outcome as well. We offer this generic and inclusive model as one step toward a deepening understanding of the human spirit that we hope will guide chaplaincy care’s continuous improvement efforts.

Models have served such purposes in other spheres. It was not until William Harvey modeled circulation in the 1600s that people were able to understand cardiovascular physiology and address illness of this system. His model was initially very simple but it allowed generation of hypotheses which resulted in experiments that refined the model until today our understanding is sufficiently sophisticated that cardiovascular conditions can be prevented and lives can be prolonged by treating its disorders. Similarly in psychology, when Sigmund Freud described transference, many others were able to refine it, and a key dynamic in psychology’s ‘physiology’ yielded a therapeutic approach that continues to evolve in various schools of psychological thought today. At a molecular level, when a model of attachment mediated by oxytocin was developed, understandings about bonding allowed more refined therapeutic approaches, including such practices as avoiding separating a mother from her newborn.

## The Chaplaincy Research Consortium model

### The setting of human spiritual experience

The first description for the model we offer is about the ‘setting’. What is the setting of the human spiritual experience? In this model we acknowledge a spiritual universe beyond the human that, for lack of a perfectly descriptive term, we refer to as the greater existence, and for which we offer the following list of words. The list is not intended to be exclusive but only illustrative. These illustrative words are not intended to favor one world view over another or to insist on a specific type of distinction between the inside world of humans and the rest of the universe. A reader not finding a word that captures the setting suitable for his or her world view is invited to add something that does.

Consciousness, Being, Love, Beauty, Oneness, God, the Unknowable, Connectedness, Timelessness, Inter-being, Presence, Transcendence, Sacredness, Holiness, the Natural World, the Universe…

This being the case, still, the model does intend to indicate that connecting to something beyond the individual is the setting for human spiritual experience.

### Spheres of human experience related to spirituality

In keeping with the model of palliative care and the domains of human suffering delineated by Cecily Saunders, we identify four domains of human experience: the physical, psychological, social, and spiritual, and we note that all of these interact with one another. We acknowledge great overlap with the work recently published by colleagues in the Netherlands that models human spirituality as embedded inside the other three domains. Our model is also distinct in some ways. These distinctions may prompt researchable hypotheses that may improve one or the other or all models; any of these would be an outcome we would welcome.

### Different ways of describing the model

The model is described in three different ways: visual, mathematical, and in a narrative of the processes of human experience. These versions are renditions of one model. It would be reasonable to use the model only in its visual or only in its mathematical or only in its narrative versions or in some combination of two or all three to generate, test, and validate or adapt different aspects of it.

## Visual depiction of the model

**Figure 1:**
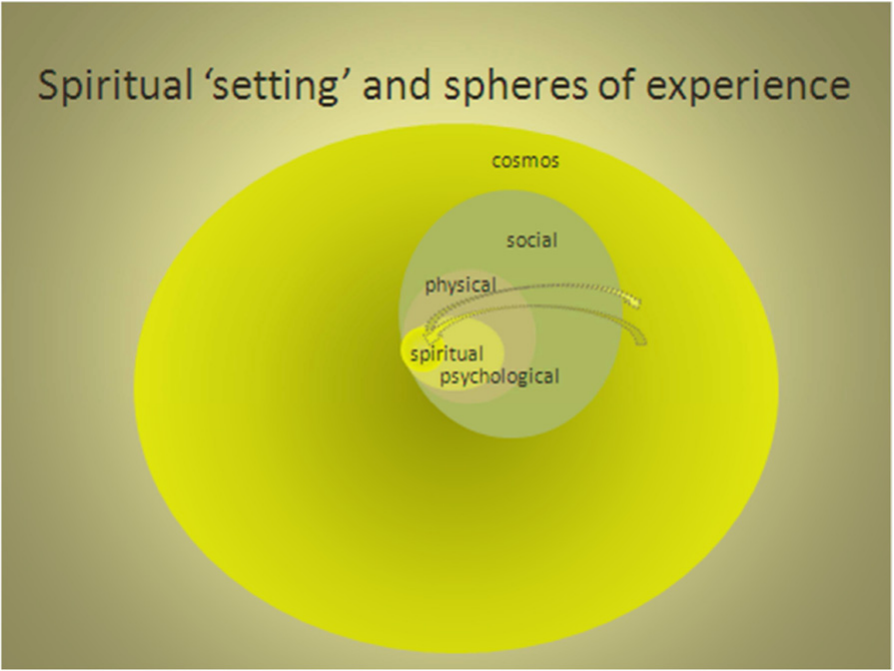
**The ‘setting’ of and relationships between the human spiritual and other domains of experience.**

We describe our model’s ‘setting’ and relationships among domains as in Figure 1 and as follows.

In our model, the individual is contained within a physical domain, within which sits the psychological and within that, at the core, is the spiritual. Boundaries between all spheres are intended to be understood as somewhat permeable. One, perhaps key, distinction is in the way our model depicts the human spiritual domain in relation to other domains. To depict the possibility of direct interaction between all spheres, each otherwise one or more layer away from the other, the spheres come up against one another in a common boundary. Lines around the spheres have been left absent to indicate the permeability of each space with respect to the other spaces.

If we could depict more than a two-dimensional figure, the largest sphere, which denotes the greater existence, would be at least three dimensional, and perhaps infinite. If the figure were larger, the social spheres would be many and variously interconnecting. Many physical individuals would exist within the social sphere, and they would have varied connections to one another. Even if all features were present and summed, these spheres would be miniscule beside the largest sphere.

Words to describe the greater existence are listed above (Sectn. 5), including dots to indicate room for more.

In the social sphere, words to depict its scope include but are not limited to the:

Dyad, Family, Neighborhood, Culture, Ethnicity, Community, City, State, Global Population…

In the physical domain we include the:

Body, Local built environment (building, neighborhood environment etc.), Natural environment (some would have this overlap with the social and psychological more fully)…

In the psychological domain we include the:

Beliefs, Attitudes, Values, Education, Aptitudes, Conscious mind, Unconscious mind, Emotions, Transference…

A feature of this model that appealed to us is the potentially direct relationship between the human spirit and the largest sphere. Direct interactions between the two are noted in most spiritual traditions, in some cases with life giving and others with cataclysmic results. Direct interactions are reported by individuals, usually with powerfully transforming effects. So our model incorporates direct interaction. This feature is, however, noted but not expanded upon since our focus is on chaplaincy and spiritual care more generally, which we regard as acknowledging but having little else to do with any direct interaction between the human spirit and the largest sphere.

Instead, of particular note for our model are the bidirectional arrows between the greatest sphere and the human spirit. These arrows have a dotted outline to indicate the possibility of interaction in all the spheres. It is the interaction between the spheres of human experience that provides material that demands and seems to create meaning-making or attribution of significance. According to our definition, spirituality engages in significance creation around the connection between the individual’s existence and the great existence. The interaction between these spheres is dynamic, and constitutes the ‘physiology’ of spirituality. This is the subject matter of the profession of chaplaincy, and this is what we seek to model in more detail now that the setting and relations between the domains is described.

## Mathematical modeling of the physiology of spirituality

A potentially useful approach to modeling is to use algebraic terms and differential equations to denote the dynamic system and its components in human spirituality.

At its simplest, the fundamental precept offers a starting equation.

$$ \mathrm{S}\kern0.2em \upalpha \kern0.2em \mathrm{f}\left(\mathrm{m}\right) $$

S = perceived spiritual state, connectedness

m = significance of spiritual place

Note that S does not denote the human spirit or the greater existence. Rather it denotes the spiritual experience as perceived by the individual. It is intended as a neutral term to designate the human spiritual experience regardless of world view. This experience is a function of the meaning or significance attributed to the experience. Two people with cancer may have very different spiritual experiences that result; one may feel abandoned and punished while the other may feel invited home. Yet a third may initially feel devastated and then come to embrace the intensity and loving connectedness of living that awareness of imminent mortality creates. The significance created by the individual, along with input from those around him or her deeply determines the spiritual experience of that individual.

### Modeling what is in the arrows

In the visual rendition of the model, the interaction between the domains of human experience was illustrated by having no boundaries drawn between the spheres and dotted boundaries in the arrows. In mathematical terms, we can use the integral function. The advantage in this is that specific factors and forms of the equation can be tried out to see what models the human experience best.

In keeping with the founding precept of palliative care and with the pictorial model of how the human spirit is situated astride and within the psychological, physical, and social domains, this equation can be further specified. If m is broken down into its components in the spiritual, psychological, physical, and social domains, the formula becomes:

$$ \mathrm{S}=\mathrm{f}\left(\mathrm{ms},\kern0.5em \mathrm{m}\uppi, \kern0.5em \mathrm{m}\upphi, \kern0.5em \mathrm{m}\upvartheta \right) $$

S = perceived spiritual experiential moment, connectedness

ms = meaning or significance in spiritual domain

mπ = sacred meaning or significance in physical domain

mϕ = sacred meaning or significance in psychological domain

mϑ = sacred meaning or significance in social domain

The formula can be further specified, depending on how the modeler of the future considers it. For instance, building on notions from the field of positive psychology in which positive and negative states can coexist, if spiritual states are considered to be a result of positive and negative attributed meaning or significance, the formula becomes:

$$ \mathrm{S}=\mathrm{f}\left(\left[\left(+\mathrm{v}\mathrm{e}\right),\left(-\mathrm{v}\mathrm{e}\right)\mathrm{m}\mathrm{s}\right],\left[\left(+\mathrm{v}\mathrm{e}\right),\left(-\mathrm{v}\mathrm{e}\right)\mathrm{m}\uppi \left],\right[\left(+\mathrm{v}\mathrm{e}\right),\left(-\mathrm{v}\mathrm{e}\right)\mathrm{m}\upphi \right],\left[\left(+\mathrm{v}\mathrm{e}\right),\left(-\mathrm{v}\mathrm{e}\right)\mathrm{m}\upvartheta \right]\right) $$

This version of the formula makes clearer that the spiritual experience can be fairly simply represented as a constructed narrative of perceived spiritual place or connectedness synthesized from the spiritual, physical, social, and psychological domains of experience. This in turn indicates something which chaplains know by experience: that the spiritual experience of a person is something that is subject to co-construction, and so to assistance by the chaplain.

An implication of this formula, which is an integral equation, is that spiritual states are about balance, about holding conflicting experiences simultaneously, and about integration. This is consistent with the views expressed by some authors such as Kenneth Pargament that spiritual suffering is about a mismatch between spiritual resources and the challenge [11].

Another important implication of this formula is that it includes a variable that is exclusively about spirituality. That is, spiritual experience (capital S) is a function of the other domains (physical, psychological, and social) as well as the spiritual domain (lower case s). This formula lends itself to empirical studies. For instance, should it be the case that the other domains can predict S alone, it could be revised with no additional variable for the spiritual domain; S would be a function of the psychological, physical, and social experiences. The new formula could then be empirically tested and the two compared.

### Modeling perturbation and chaplaincy co-construction

Chaplains are often called upon in a palliative care setting because the patient’s illness has prompted a perturbation. The prior worldview that functioned well enough is no longer understood as consistent with the facts [13]. Perhaps the patient has, with or without necessarily articulating it, functioned with a worldview that had a benevolent and purposeful place for his or her existence. Facing mortality makes the prior worldview seem no longer benevolent or purposeful, perhaps. The balance of significant information coming from the physical sphere (previously all positive: all is well; you are healthy and alive) has been changed by the negative information (this illness will likely kill you) and is not consistent with the prior psychological information (you are safe) and spiritual information (your life has a place that matters) which therefore has been challenged: there is a frightening likelihood of being not safe and life being insignificant. Perhaps the earlier equilibrium is also inconsistent with the social information (perhaps people are withdrawing or relationships are changing as loved ones prepare themselves for bereavement). The person’s state, S1, had changed to S2 in which the negative values now trump the positive values.

$$ \mathrm{S}1=\mathrm{f}\sum \left(\mathrm{large} + \mathrm{v}\mathrm{e}\right)-\left(\mathrm{small}-\mathrm{v}\mathrm{e}\right)\mathrm{m}\mathrm{s},\left(\mathrm{large} + \mathrm{v}\mathrm{e}\right)-\left(\mathrm{small}-\mathrm{v}\mathrm{e}\right)\mathrm{m}\uppi, \left(\mathrm{large} + \mathrm{v}\mathrm{e}\right)-\left(\mathrm{small}-\mathrm{v}\mathrm{e}\right)\mathrm{m}\upphi, \left(\mathrm{large}+\mathrm{v}\mathrm{e}\right)-\left(\mathrm{small}-\mathrm{v}\mathrm{e}\right)\mathrm{m}\upvartheta $$

$$ \mathrm{S}2=\mathrm{f}\sum \left(\mathrm{small}+\mathrm{v}\mathrm{e}\right)-\left(\mathrm{large}-\mathrm{v}\mathrm{e}\right)\mathrm{m}\mathrm{s},\left(\mathrm{small}+\mathrm{v}\mathrm{e}\right)-\left(\mathrm{large}-\mathrm{v}\mathrm{e}\right)\mathrm{m}\uppi, \left(\mathrm{small}+\mathrm{v}\mathrm{e}\right)-\left(\mathrm{large}-\mathrm{v}\mathrm{e}\right)\mathrm{m}\upphi, \left(\mathrm{small}+\mathrm{v}\mathrm{e}\right)-\left(\mathrm{large}-\mathrm{v}\mathrm{e}\right)\mathrm{m}\upvartheta $$

The chaplain then has the task of helping the patient find ways of understanding the meaning or significance of cancer and its consequences. Perhaps it involves understanding that the person’s life matters despite or even because of mortality, that dying may be hard but it will be eased by palliative care and death itself is not fearsome, and that preparation for bereavement is something the patient wants for his or her loved ones. For many people, it involves adjusting their understanding of their relationship with their higher power. By helping to adjust the attributed significance in each of these domains, the patient’s integrated perception can reach a new equilibrium. Sometimes this new state of understanding is considerably more wholesome than the earlier one and can bring a peaceful transformation to the patient and her or his loved ones. Note that, in its above form, this mathematical model assumes that transformation is the product of a new balance. It also does not have a time factor in the model and in this sense assumes that new balances can occur over any time period. This is taken up further below.

People engage in many types of coping. As is described below, some types of spiritual coping yield greater engagement and others yield temporary or entrenched disengagement. One person may pray or listen to music more, another less. The same person may go through phases. Other coping mechanisms may kick in and be used more or less, depending on the person or the time. In mathematical terms it might be best modeled as a waveform.

The arrow indicates an assault (e.g. a new diagnosis) to the system of interpreted or narrative significance. After the perturbation, the new equilibrium, if it is achieved, may be similar to the prior state or at a higher or lower level.

Understanding perturbation in this way introduces another necessary development of the mathematical model: time.

The equation becomes:

$$ \mathrm{S}=\underset{\mathrm{t}=\mathrm{n}+1,2,\dots }{\overset{\mathrm{t}=\mathrm{n}}{f\sum }}\left(\left[\left(+\mathrm{v}\mathrm{e}\right),\left(-\mathrm{v}\mathrm{e}\right)\mathrm{m}\mathrm{s}\right],\left[\left(+\mathrm{v}\mathrm{e}\right),\left(-\mathrm{v}\mathrm{e}\right)\mathrm{m}\uppi \right],\left[\left(+\mathrm{v}\mathrm{e}\right),\left(-\mathrm{v}\mathrm{e}\right)\mathrm{m}\upphi \right],\left.\left(+\mathrm{v}\mathrm{e}\right),\left(-\mathrm{v}\mathrm{e}\right)\mathrm{m}\upvartheta \right]\right) $$

Time is an interesting feature to add to the equation partly because it is clearer and more open to exploration than trying to put it in the visual version of the model, which would have to become animated to display time. It is also interesting because people describe time as unusual in relation to spiritual experience. It can be fleeting but feel like an eternity, for example. Research on subjective experiences of time in spiritual matters could substantiate a distinctive time perception in this sphere.

Researchers may use this mathematical model to hypothesize what belongs in the positive or negative categories in each domain for which populations or what type of experience takes what time range to equilibrate, etc. Then it becomes more manageable to design ways to test the hypotheses in empirical settings.

## Narrative description of spiritual processes

The mathematical model depicts interacting components but not processes or mechanisms by which they interact. A greater understanding of what happens in the processes of the human spiritual ‘apparatus’ is provided by Pargament’s model of discovery, conservation, and transformation, which, with some adaptation, we adopt. As presented here, the states are four, and the movement between states is unconstrained. Spiritual processes entail movement between these states.

Our model’s presumption is that this movement between 4 states describes the wave of perturbation, and is how the integration (meaning making or significance attribution) among the factors (spheres of experience) in the mathematical (or visual) depictions of our model occurs. In the visual depiction, the movement between these four states can be considered a magnified rendition of what happens in the arrows that travel between the spheres of experience.

### Four-step recursive processing of spiritual experience

The human spirit engages in a recursive process entailing four distinct states and distinct types of outcome. All states can lead to any other state in this model and there is not an irrevocable one way movement or an exclusive starting point for spiritual experience.

**Figure 2:**
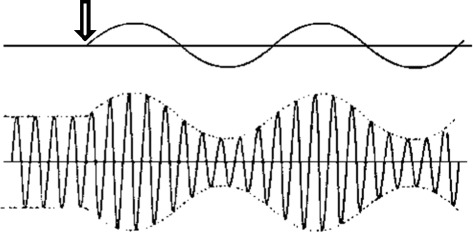
**Perturbation and spiritual coping.**
  Research may reveal that this state is well recognized but little researched in clinical practice. Clinicians routinely acknowledge that when a patient is facing new terrible news they become refractory to intake of new information. For instance, a patient learning s/he has cancer does not take in any other information offered by the clinician for a while, something that may be difficult if immediate treatment decisions are needed. Chaplains are also alert to this circumstance for a different reason. This is the ‘window’ of time when his or her presence may be particularly important. Future research may inquire whether there are physical (neurological or endocrine perhaps) mediators of these state when cognitive function is arrested and spiritual frames shift. Future research may delineate how this process impacts decision-making and thence guide clinical teams and family members how to support the process best.
- Dialogue:
  With the discovery of a frameshifting connection, the person is usually motivated to engage in the relationship with the greater existence or to engage the changed worldview. Some may engage in a Buberian type of intimate I-thou sharing, which may occur prayerfully or meditatively, by reading, or in dialogue with people. Some may have a heightened sense of the sacredness in many things, and the dialogue may not be verbal or cognitive but interactively experiential in some other way. But the step is exploratory, questioning, and seeking of coherence.
  During the dialogue stage, a person can strengthen or conserve (the term ‘conservation’ is used for this step in Pargament’s model) the spiritual resources that he or she can access, gathering ease with gleaning spiritual ‘nutrition’ or connectedness. The purpose of the dialogue is to weave together the meaning or significance of this new connection with the sacred with other aspects of life and mortality, to create something coherent and integrated that works to achieve spiritual wellness. For some, the dialogue results in conserving the pre-discovery equilibrium; for others a new state is reached.
- Struggle:
  Alternatively, the engagement may not be nurturing and positive; for many it is a fearful engagement, or one of pleading and bargaining and great anxiety. And new stages can be better or worse. The dialogue may not strengthen spiritual resources, but rather leave the person feeling fragmented, confused and abandoned, receiving silence when meaning or significance was needed, and sensing there is nowhere safe to turn except perhaps nothingness. A person’s spiritual search may oscillate between more negative and more positive states in any or all of the spheres of experience, or it may be largely negative and the waveform of experiences may be chaotic and unbalanced, and so on. To allow characterization of these states of spiritual suffering, we distinguish a separate state: spiritual struggle.
  Spiritual challenges, like challenges in other spheres, can be characterized as a balance between spiritual needs and spiritual resources. The fundamental need, by our definition, is for connection with the greater existence. How that connection occurs varies widely, however. Help with conservation, returning to a state of dialogue, can occur by bolstering resources that work for the individual. A favorite psalm or manageable activity such as fishing that brings them a sense of sacredness can help. Somehow, finding coherence with the greater existence into which their whole life can meaningfully and comfortably fit is what brings resolution to the struggle.
  Failure to find this can result in efforts to connect that have a negative spiral. Efforts to dampen the suffering by numbing sensations with alcohol or emotional detachment tend to seal a person off from resources. Withdrawal and depression can accelerate. Anxiety can result in sleep deprivation and social isolation that limits access to resources and can result in fragmentation. Seeking intimacy in violent encounters or transformation through stimulant chemicals tends to trigger law enforcement consequences with guilt, shame and other social ruptures that also lock into cycles that spin out. All these forms of spiritual suffering have the risk of acceleration until the person has reached ‘rock bottom’. Far from the reaches of personal and social support, it is classic that a person who does bounce back does so with the experience of a direct connection with the greater existence.
- Arrival/disconnection:
  The conservation or dialogue stage and the struggle or suffering state eventually result in a new stage or state. Transformation is the term used in Pargament’s model. This state is an outcome state. Two main outcome types result: one is of feeling integrated and at peace, the other is of alienation and disconnection. While this model describes a new state that has one of two dichotomous possibilities, for many people there is no dramatic transformation but rather a steady state or gradual evolution, and for many people the states of peaceful integration and alienated disconnection co-exist in proportions that are part of a continuum. Nonetheless, the model distinguishes states of being that people grapple with and their identification in words is helpful.
  A stage or state of integration and peace entails understanding one’s situation in a way that is harmonious with global or universal meaning or the greater existence. It goes along with an infectious sense of love, peace, beauty, and presence, including when terminally debilitated.
  In the clinical world of palliative care chaplaincy, a state of alienation and disconnection may be considered the equivalent to an adverse event and an emergency. This is ultimate spiritual suffering. Presence, being heard, and normalizing the person’s experience, seem to mitigate the state and help a return to more successful struggles and transition to the state of conservation or dialogue. Sometimes it is in the state of alienation and disconnection that a person experiences the direct connection to the greater existence.
  As chaplains and others observe, these states may be variably engaged, either by disposition or by practice, and the practices of each step may further spiritual qualities that are more or less helpful to achieving desired spiritual states of being. Some seem to be ‘born spiritual’ and those who engage deeply in their spiritual life seem to develop strengths and spiritual capacities akin to those in the physical or other domains that respond to training and practice. Others may have more difficulty accessing spiritual resources for themselves.
  For empirical researchers, this aspect of our model is of interest because it points to two types of outcome measure: integration and peace or alienation and suffering. One measure “Are you at peace” developed by Steinhauser et al. [14] may correspond to the former. Whether the latter is simply lack of integration and peace or on a different scale altogether is an important empirical question. Indeed, all of these states are open to empirical validation or adjustment based on findings.

- Discovery:
  In the stage of discovery, a person experiences sacredness. In our model, the experience may be in one form or another but it is an experience involving a connection to the greater existence. It may be that the person senses his or her finitude, and the feeling of vulnerability opens the person to the possibility of experiencing sacredness. The trigger may be unwanted (such as a new illness or a recurrence or worsening illness). For the palliative care chaplain this type may be the commonest situation. Or it may happen in another way, such as an experience of beauty or new life (such as watching eggs hatch).
  We acknowledge that this stage of discovery may occur in the direct connection to the greater existence, as noted above and as seen in Figure 1 where we depict the spiritual sphere against the greater existence sphere free of other intervening spheres at one point along the boundaries. This discovery moment is an experience about being and becoming or something closely related to that. Above, we note that chaplains understand but do not attempt to generate such direct interaction, which could be hubris. At the same time, we also acknowledge that by working with the creation of significance in the psychological, physical, social and spiritual spheres, using ritual, prayer, or other interventions, the chaplain may help a person to create an integrated disposition in which direct connection can be recognized or sought and perhaps occur more readily.
  We also acknowledge that this stage can occur in its converse form. A person can feel separated from the sacred, feel nothingness where presence was possible or expected, or abandonment where love was sought. In the model, this state has positive and negative renditions and may be multivalent.
  However it happens, the result of discovery is a new round of processing and the potential for reaching a new state of equilibrium exists. This discovery state relates to the arrow in Figure 2 that represents a shock to the system in that discovery can be a perturbation stimulus to the system and the subsequent rounds of processing depicted by oscillating wave forms are described by the next steps.

### Getting from step to step: interventions to care for the human spirit

Understanding how a person moves from step to step (discovery, dialogue, struggle, and arrival or disconnection) in each of the spheres of experience, and especially in the spiritual sphere is a part of the model not yet well delineated and inviting of further description. This is critical to understanding how care might work best, and it can come from empirical work.

### Studying listening, witnessing, and other care skills

One of the skills of chaplains and others who care for the human spirit is listening. Research might discover that listening is a critical component of the dialogue step. Empirical work could establish that some forms of listening are more effective than others. Another healing interaction can come in the form of witnessing or acknowledgement or silent presence, whether in a dyad or a group. Research might discover that this is a critical component of transitioning from struggle to arrival or of reversing disconnectedness. Such findings could sort out which of the interventions currently used fits best for specific needs and stages of a person.

### The fabric of processing

**Figure 3:**
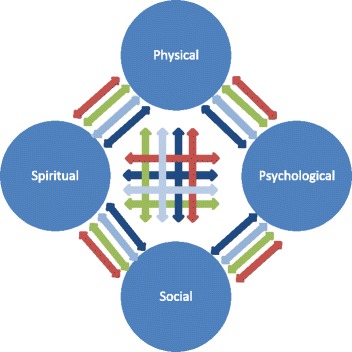
**Fabric of Processing.** Four stages, each depicted by different colors, of processing –  discovery,  dialogue,  struggle, and arrival or disconnection – to create integrated meaning or significance.

Nonetheless, even before all this empirical work is done, we can suggest that all four of the above steps in spiritual processing (discovery, dialogue, struggle, arrival/disconnection) can occur in relationship between any of the four spheres of experience (physical, social, psychological, and spiritual) since spiritual features related to all of them. This would suggest a visual rendition that we have termed the ‘Fabric of Processing’, as depicted in Figure 3. This fabric model is an integration of the four-step recursive process with the interactions depicted by the bidirectional arrows in the overall visual model in Figure 1, and an expanded representation of what exists in the integral function in the mathematical model.

A characteristic of the model is the possibility of transformation and breakthrough. Equally, however, the model describes arrival at the same kind of outcomes through accumulations of experience in the everyday spheres of psychology, physical and social experience that can yield a strong sense, that works for people, for the sacred. While spiritual transformation is unique to its sphere, we also note that analogous transformation occurs in other spheres. In the psychological sphere, it is well accepted that attitudes can change in a moment and alter a person’s life. In human development, transitions can be so profound that they are considered transformations. In the social spheres cultural changes can be radical and rapid such that they are considered revolutions or social transformations. Even in the physical sphere metamorphoses occur both naturally and medically; in the latter an example might be when a missing or replaced hormone can transform a person from the brink of death to perfect health or vice versa. Transformation is considered an integral part of our spirituality model but not a necessary means to arrive at spiritual peace.

### The key movement

In addition to describing the states entailed in spiritual processes it is helpful to identify a key movement. In our model, the key movement is *connecting*. The connecting may be particular, but its significance is in relation to the sacred. It is received as a sense of belonging, peace, love, and beauty. Or the obverse sensations happen when the connecting is not working. Notably, the connecting may occur through so many different vehicles that recognizing it as such may be elusive. As described in the definition of sacredness, people experience connectedness to the greater existence in ways as varied as returning to a long standing bridge game group or sky diving, listening to Tibetan music or performing brain surgery.

The importance of saying that our model in Figure 1 would depict the greater existence sphere as at least three dimensional may make especial sense here. The connecting can happen in essentially any setting – a prison in Pretoria or overlooking the Niagara Falls – and any domain of experience – psychological, physical, social, or directly to the spiritual.

Importantly for the model and its use in helping chaplaincy, a second key movement is *integrating*. This integration seems to be greatly but not uniquely connected to meaning making or attribution of significance and may very well be facilitated by connecting. At a fully integrated state, the experience appears to be one of peace, or poise, even in the setting of great change or chaos.

## Using the model

### Hypotheses

The main purpose of the model is to provide a working understanding of the ‘physiology’ of the human spirit in palliative care settings so that it can aid in the generation of hypotheses for study and aid in assessing the state of research so that gaps in how care of the human spirit is provided can be more readily noted and responded to [15-17].

Similarly, the validity of the model can be somewhat assessed by mapping on to it existing studies to assess the goodness of fit with the way researchers are asking questions about human spirituality.

### Measuring spiritual states - direct or as part of other domains

Hypotheses generated by the model cannot be evaluated without adequate measures. At the same time, guidance can be taken from the model about how to approach creation of measures [18].

For instance, spiritual states appear to be expressed in all three of the other domains. Spiritual equanimity seems to correlate with psychological peacefulness (e.g. low anxiety, positive mood), physical equilibrium (e.g. good blood pressure, fewer infections and immune disorders), and social ease (e.g. pleasant levels of social engagement, few quarrels). Spiritual suffering seems to correlate with the opposite (anxiety and depression, high cortisol levels and social discord). Recent studies and a review of literature on social isolation indicated that it is correlated with many disorders that entail suffering and shortened life [19,20]. This review’s gathered findings cohere with our model’s precept that humans have a fundamental need to connect to one another that is analogous to other essential ingredients such as water and carbohydrates that sustain life. Additional recent work has indicated how sharing of deeply meaningful experiences can share their impact. Holocaust survivors telling their stories had reduced biomarkers of stress, while those who heard the stories had rising biomarkers of stress. These findings indicate that measures of spiritual state can be found among physical biomarkers and by using psychometric and social metrics. One hypothesis from this may be that spiritual wholeness, wellness, or integrity also needs a basic sense of connectedness. However, we note that our model could generate this hypothesis but does not depend on it; it is open to empirical findings about human experiences.

Direct measures of spiritual states also exist, such as Steinhauser’s above noted one item measure “Are you at peace?”, FACIT-Sp, SDAT and others [14,21,22]. Measurement sciences, particularly Rasch analysis, may be able to help assess which states are distinctive and which are part of an underlying state (latent state in Rasch terminology). Since FACIT and SDAT have multiple items (12 and 5 respectively) and each have two subscales (meaning and faith for FACIT, and intrinsic and extrinsic for SDAT), a comparison between these measurement constructs and the model elements may be helpful. So, for instance, further work, perhaps on a construct and then a measure for spiritual suffering or for the dialogue or discovery states, may be able to validate or refute, or adjust the posited states in this model. Especially the one-item measure seems likely to be a good way to assess to what degree a person has achieved at least a major component of or the overall ‘arrival’ state.

### Evolution of the model

In the end, the model must be able to evolve in response to empirical findings. A test of the quality of this model will be how well it evolves over time. We look forward to many empirical studies that will result in its evolution.
